# Valedictory

**Published:** 1852-03

**Authors:** 


### Part 4.—Editorial.

### ARTICLE I.

### VALEDICTORY.

After laboring six years in the editorial department of the Journal, it might be expected that we should, on retiring from the post, write an extended valedictory address. But we have during our connection with the Journal, expressed our views freely and independently upon all subjects upon which we have been called upon to write, and where they have been correct, they need no comment; where they may have been erroneous, we feel satisfied that no renunciation at this late period will set them right. We have but a poor opinion of last confessions at any rate, so we shall take leave of our tripod and numerous friends without any rehearsal of the past.

We feel a satisfaction in leaving the interests of the Journal in able and experienced hands, and hope that the prosperity that has attended it during our labors will be continued even more abundantly.

The terms and condition upon which the Journal will hereafter be published, may be found in the Prospectus of the next volume on the cover.

We take leave of our readers, though personally unacquint ed with many of them, with regret, as there is a satisfaction in constantly communicating, that we are sorry to lose.

Page(s) missing

To the numerous contributors to our pages, we return our sincere thanks for the valuable and indispensable aid they have given us, and bespeak for our successor and for the profession at large, that they will continue their able and interesting communications.

With our cotemporaries of the editorial fraternity, for whose numerous marks of kindness and attention we are under obligations, we part as with brethren of a sacred tie; hoping that they each and all may have the satisfaction of seeing the good work of enlightening the profession prosper in their hands.

